# National U.S. Patient and Transplant Data for Krabbe Disease

**DOI:** 10.3389/fped.2021.764626

**Published:** 2021-11-11

**Authors:** Gabrielle Ghabash, Jacob Wilkes, Joshua L. Bonkowsky

**Affiliations:** ^1^University of Utah School of Medicine, Salt Lake City, UT, United States; ^2^Intermountain Healthcare, Salt Lake City, UT, United States; ^3^Division of Pediatric Neurology, Department of Pediatrics, University of Utah School of Medicine, Salt Lake City, UT, United States; ^4^Primary Children's Center for Personalized Medicine, Primary Children's Hospital, Salt Lake City, UT, United States

**Keywords:** leukodystrophy, Krabbe disease (globoid cell leukodystrophy), pediatric neurology, hematopoietic stem cell transplantation, disparities (health racial)

## Abstract

Krabbe disease (KD) is a leukodystrophy caused by mutations in the *galactosylceramidase* gene. Presymptomatic hematopoietic stem cell transplantation (HSCT) is associated with improved outcomes, but most data are from single-center studies. Our objective was to characterize national patterns of HSCT for KD including whether there were disparities in HSCT utilization and outcomes. We conducted a retrospective study of KD patients ≤ age 18 years from November 1, 2015, through December 31, 2019, using the U.S. Children's Hospital Association's Pediatric Health Information System database. We evaluated outcomes for HSCT, intensive care unit days, and mortality, comparing age, sex, race/ethnicity, rural/urban location, and median household income. We identified 91 KD patients. HSCT, performed in 32% of patients, was associated with reduced mortality, 31 vs. 68% without HSCT (*p* < 0.003). Trends included the fact that more males than females had HSCT (39 vs. 23%); more Asian and White patients had HSCT compared to Black or Hispanic patients (75, 33, 25, and 17%, respectively); and patients from households with the lowest-income quartile (< $25,000) had more HSCT compared to higher-income quartiles (44 vs. 33, 30, and 0%). Overall, receiving HSCT was associated with reduced mortality. We noted trends in patient groups who received HSCT. Our findings suggest that disparities in receiving HSCT could affect outcomes for KD patients.

## Introduction

Krabbe disease (KD) is a neurodegenerative leukodystrophy caused by mutations in the *galactosylceramidase* (*GALC*) gene, which encodes the lysosomal b-galactocerebrosidase enzyme involved in myelin turnover (1). The clinical phenotype of KD is variable, depending on the molecular mutation type in *GALC*, the amount of decrease in enzyme activity, and the degree of elevation of psychosine, a toxic metabolite generated by deacylation of galactosylceramide (2–4). Early infantile onset KD is the most common as well as the most severe form, characterized by symptom onset prior to 6 months and an average life span of <3 years, and accounts for >80% of all KD cases (5, 6). Current treatment for KD is hematopoietic stem cell transplantation (HSCT), which is effective only if performed prior to symptom onset (7–10).

The potential for treatment with HSCT and the development of novel therapies including gene therapy (11, 12) has led to efforts to increase early identification of KD patients, including 10 states that have implemented KD newborn screening (NBS) (13–15). However, it is not known whether treatment, outcomes, and mortality for KD are affected by disparities including socioeconomic, racial, or gender background, and which thus might impact efforts for testing and identification of patients. A recent study identified underdiagnosis of KD and other leukodystrophies in Black and Hispanic children (16). Furthermore, most current data on KD treatment and outcomes have come from a few centers, and it is unknown whether the outcomes for KD patients are similar across the entire U.S. Our objective was to evaluate KD in a national analysis of data, focusing on HSCT use, outcomes, and mortality, including whether there were health outcome disparities for KD patients.

## Materials and Methods

### Standard Protocol Approvals, Registrations, and Patient Consents

This project using deidentified data was not considered human subjects research in accordance with the Common Rule [45CFR§46.102(f)] and was exempted by the Institutional Review Boards at the University of Utah and Intermountain Healthcare.

### Study Design

We conducted a retrospective study on patients ≤ age 18 years admitted to a Pediatric Health Information System (PHIS) hospital with an International Classification of Diseases, Tenth Revision (ICD-10-CM) diagnosis of KD (E75.23), with an admission date between November 1, 2015, through December 31, 2019. A unique ICD code for KD did not exist prior to implementation of ICD-10-CM in October 2015. Thirty-two individuals who had a date of first admission prior to November 1, 2015 were not included in the study. Additionally, three individuals were over the age of 18 on their date of first admission and were dropped from analyses. Year of birth spanned from 1997 to 2019 and dates of first admission ranged from November 1, 2015 to October 15, 2019.

The PHIS database has information from 50+ children's hospitals in the U.S. (15). Hospitals are affiliated with the business alliance of Child Health Corporation of America (Shawnee Mission, Kansas). Each visit has a unique entry and is linked to a unique patient, but the data are deidentified. Data collected includes patient and visit demographics such as age, gender, race, Rural–Urban Commuting Area (RUCA) codes, insurance type, type of visit, estimated total visit cost, length of stay, and ICD coding and detailed charge information. The hospital visits included primarily inpatient admissions with additional visit types such as emergency department, observation, ambulatory surgery, clinic, and other visit types where available. The R package *dplyr* (17) was used to compress all visits by medical record number.

### Statistical Analysis

Statistical analyses were conducted using R v.4.0.2^1^. Plots and descriptive table were generated using *ggplot2* and *table1* (www.rdocumentation.org/packages/table1; downloaded April 10, 2020) packages, respectively (18)^2^.

All analyses were two-sided and *p* < 0.05 was considered statistically significant. Chi-squared tests were used for categorical data where the number of individuals per bin was >5. Two-tailed Welch *t*-tests and analysis of variance (ANOVA) were used to analyze differences between groupings.

### Clinical Course and Characteristics

#### Transplant Status

The presence of hematopoietic stem cell transplant (HSCT) (“transplant”) and patient age at HSCT were collected. HSCT status was determined by the presence of at least one of the following ICD-10 diagnoses: bone marrow transplant status, stem cell transplant status, complications of stem cell transplant, and encounter for aftercare following bone marrow transplant (Z9481, Z9484, T865, and Z48290); ICD-9 diagnoses for bone marrow transplant status and peripheral stem cells replaced by transplant (V4281, V4282); ICD-10 procedure codes for transfusion of non-autologous cord blood stem cells into central vein percutaneous approach, transfusion of allogeneic unrelated cord blood stem cells into central vein percutaneous approach, transfusion of allogeneic unrelated hematopoietic stem cells into central vein percutaneous approach, transfusion of allogeneic unspecified cord blood stem cells into central vein percutaneous approach, transfusion of allogeneic unspecified cord blood stem cells into peripheral vein percutaneous approach, transfusion of autologous hematopoietic stem cells into central vein percutaneous approach, and transfusion of allogeneic related hematopoietic stem cells into central vein percutaneous approach (30243X1, 30243X3, 30243Y3, 30243X4, 30233X4, 30243Y0, and 30243Y2); ICD-9 procedure codes for allogeneic HSCT without purging, cord blood stem cell transplant, and bone marrow transplant not otherwise specified (ICD-9 4105, 4106, and 4100). The discharge date corresponding to the first appearance of any one of these diagnoses or procedures was denoted as the date of transplant and age of transplant was calculated in years. Age of transplant was grouped by age: <6 months, 6–36 months, and after 36 months of age, corresponding to Early Infantile, Late Infantile, and Later Onset forms of KD (19, 20).

#### Mortality

PHIS reports in-hospital deaths only; five were recorded. To more fully estimate mortality, if a patient did not have an entry in PHIS for >1 year after their last visit, they were assumed deceased.

#### Additional Characteristics

In addition to presence of transplant, data on age at transplant and mortality, age at first admission, age at last discharge, length of follow-up, and number of ICU days were collected. Age at first admission was grouped by <6 months, 6–36 months, and >36 months of age, corresponding to Early Infantile, Late Infantile, and Later Onset forms of KD. Age at last discharge was grouped by less than or greater than 36 months, corresponding to 12 months greater than the accepted life expectancy after symptom onset for Early Infantile KD (19).

### Groupings

#### Race

In PHIS, race and ethnicity are collected as patient or family-reported answers to separate questions at registration: one for race and one for ethnicity. Individuals who reported White-Non-Hispanic and White-Hispanic at different visits were categorized as Hispanic.

#### Insurance

PHIS provides information on the payer for each visit. The most frequent payer per individual was determined. In cases where multiple different payers covered the same number of visits for an individual, the payer at first visit was used. Categories of Public, Private, and Other were defined. Public included in-state Medicaid, out-of-state Medicaid, and Children's Health Insurance Program (CHIP). Private included commercial insurance only. Other included cases of multiple insurance types, TRICARE, self-pay, or no charge.

#### Rural–Urban Commuting Area Codes

PHIS provides RUCA codes^3^ based on patient home zip code, which contains information about population density based on 2000 census data^4^. The most frequent RUCA score for each patient was determined. A RUCA score of 1.0, 2.0, or 3.0 was defined as urban; 4.0, 5.0, or 6.0 as micropolitan; 7.0, 8.0, or 9.0 as small rural town; and 10.0 as isolated small rural town.

#### Median Household Income

The most frequent annual median household income determined by zip code for each individual was grouped by < $25,000, between $25,000 and $50,000, between $50,000 and $75,000, and >$75,000 per year. In cases where different incomes were reported the same number of times, the income at first visit was used.

### Data Availability Statement

All data presented and reported are included in this manuscript.

## Results

**Figure 1:**
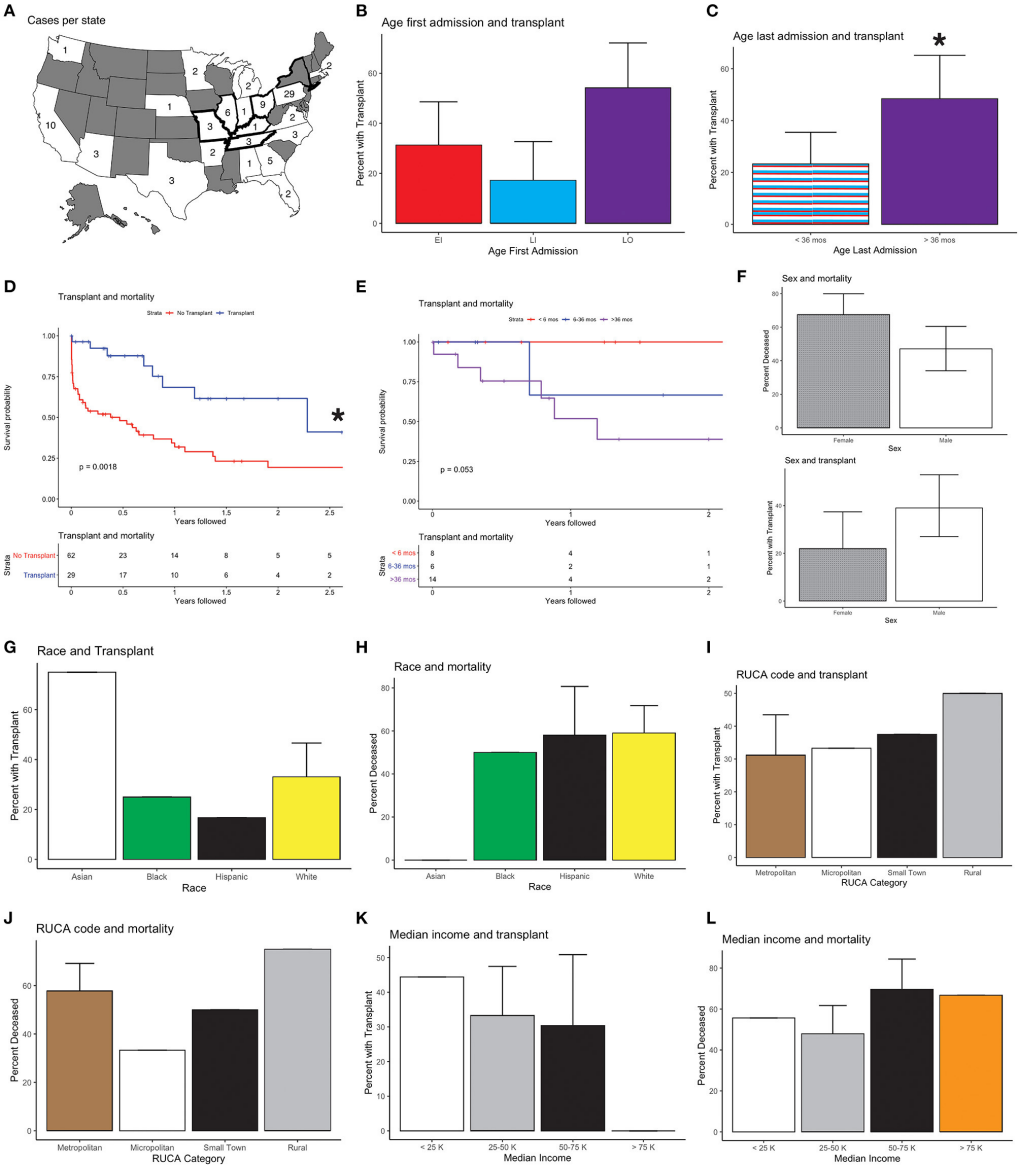
National Krabbe disease (KD) data and outcomes; 95% confidence intervals are shown. **(A)** Cases per state. Bolded outlines denote states with KD on their newborn screening panel during the study period. **(B)** Age at first admission and transplant (HSCT) status. Age at first admission was correlated with receiving HSCT, with patients who presented after 36 months being more likely to receive HSCT than individuals who first presented between 6 and 36 months of life (*p* < 0.02). All other comparisons, EI vs. LI and EI vs. LO, were not significant (*p* > 0.1). **(C)** Age of last admission and HSCT status. Age at last admission >36 months was associated with HSCT (*p* < 0.03). **(D)** Kaplan-Meier survival curves of mortality and HSCT. Mortality and absence of HSCT were correlated (*p* < 0.01). **(E)** Mortality and age of HSCT. One hundred percent (*N* = 8) of individuals who received HSCT prior to 6 months, 67% (*N* = 4) of individuals who received transplant between 6 and 36 months, and 50% (*N* = 7) of individuals who received transplant after 36 months were determined to be alive (*p* > 0.05). **(F)** Sex and transplant. More males than females had HSCT (56 vs. 44%) (*p* > 0.08), and a higher fraction of females were deceased compared to males after HSCT (67.5 vs. 47%) (*p* = 0.0503). **(G)** Race and transplant. Seventy-five percent (*N* = 3) of Asian, 25% (*N* = 2) of Black, 17% (*N* = 2) of Hispanic, and 33% (*N* = 16) of White individuals received HSCT (*p* > 0.3). **(H)** Race and mortality. Fifty-nine percent (*N* = 21) of White, 58% (*N* = 7) of Hispanic, 50% (*N* = 4) of Black, and 0% (*N* = 4) of Asian patients were found to be deceased (*p* > 0.1). **(I)** Rural–Urban Commuting Area (RUCA) Code and transplant. Thirty-one percent (*N* = 20) of individuals in urban areas, 33% (*N* = 2) of individuals in micropolitan areas, 37.5% (*N* = 3) of individuals in small rural town, and 50% (*N* = 2) of individuals in isolated rural areas received HSCT (*p* > 0.8). **(J)** RUCA and mortality. Fifty-eight percent (*N* = 37) of individuals in urban areas, 33% (*N* = 2) in micropolitan areas, 50% (*N* = 4) in small rural town, and 75% (*N* = 3) in isolated rural areas were classified as deceased (*p* > 0.5). **(K)** Median household income and transplant. Forty-four percent (*N* = 4) of individuals with income <25,000, 33% (*N* = 16) of individuals with income between 25,000 and 50,000, 30% (*N* = 7) with income between 50,000 and 75,000, and 0% (*N* = 0) of individuals with income >75,000 received HSCT (*p* > 0.5). **(L)** Median household income and mortality. Fifty-six percent (*N* = 5) of individuals with income <25,000, 48% (*N* = 23) of individuals with income between 25,000 and 50,000, 70% (*N* = 16) with income between 50,000 and 75,000, and 67% (*N* = 2) of individuals with income >75,000 were presumed to be deceased (*p* > 0.3). *Statistically significant (*p* < 0.05).

We analyzed all infants and children (birth through age 18 years) admitted to a PHIS hospital between November 1, 2015 and December 31, 2019 with an International Classification of Diseases, Tenth Revision (ICD-10-CM) diagnosis of KD (E75.23). Selected demographic and clinical descriptors of the 91 identified KD patients are summarized in Table 1; their geographic distribution is shown in Figure 1A.

**Table 1:** Selected demographics of KD cohort.

**Characteristic**	**All (*N* = 91)**	**No HSCT (*N* = 62)**	**HSCT (*N* = 29)**	***p^**e**^***
**Age first admission (years)**				>0.07
Mean (SD)	2.4 (3.6)	1.9 (3.1)	3.5 (4.3)	
Median [Min, Max]	0.83 [0.0, 17,7]	0.77 [0, 17,7]	2.3 [0.0, 14.9]	
**Age at last follow-up (years)**				<0.05
Mean (SD)	3.1 (3.8)	2.5 (3.4)	4.4 (4.4)	
Median [Min, Max]	1.5 [0.06. 21.2]	1.2 [0.06, 21.2]	3.3 [0.28, 17,2]	
**Length of follow-up (years)**				>0.2
Mean (SD)	0.72 (0.95)	0.64 (1.0)	0.89 (0.80)	
Median [Min, Max]	0.35 [0.0, 3.9]	0.17 [0.0, 3.9]	0.69 [0.0, 2.9]	
**ICU days**				>0.3
Mean (SD)	4.6 (15.5)	3.1 (8.0)	7.8 (24.8)	
Median [Min, Max]	0 [0, 130]	0 [0, 41]	0 [0, 130]	
**Mortality**				<0.003
Deceased	51 (56%)	42 (68%)	9 (31%)	
Alive	42 (44%)	22 (32%)	20 (69%)	
**Sex**				>0.08
Female	40 (44%)	31 (50%)	9 (31%)	
Male	51 (56%)	31 (50%)	20 (69%)	
**Race/ethnicity** ^**a**^				>0.3
Asian	4 (4%)	1 (2%)	3 (10%)	
Black	8 (9%)	6 (10%)	2 (7%)	
Hispanic	12 (13%)	10 (16%)	2 (7%)	
White	49 (54%)	33 (53%)	16 (55%)	
**Median household income** ^**b**^				>0.5
<25 K	9 (10%)	5 (8%)	4 (14%)	
25–50 K	48 (53%)	32 (52%)	16 (55%)	
50–75 K	23 (25%)	16 (23%)	7 (24%)	
>75 K	3 (5%)	3 (5%)	0 (0%)	
**Insurance** ^**c**^				>0.5
Private	35 (38%)	21 (34%)	14 (48%)	
Public	42 (46%)	29 (47%)	13 (45%)	
**Rural–urban commuting code category (RUCA)** ^**d**^				>0.8
Urban	64 (70%)	44 (71%)	20 (69%)	
Micropolitan	6 (7%)	4 (7%)	2 (7%)	
Small rural town	8 (9%)	5 (8%)	3 (10%)	
Isolated rural town	4 (4%)	2 (3%)	2 (7%)	

*HSCT, hematopoietic stem cell transplantation*.

a *Eighteen individuals had multiple or unknown race/ethnicity*.

b *Eight individuals had missing income data*.

c *Fourteen individuals had Other insurance, including TRICARE, self-pay, and no charge*.

d *Nineteen had missing RUCA codes*.

e *Two-tailed t-test; except for chi-squared (Mortality, Sex, Income, and Insurance), or ANOVA followed by Tukey HSD (Race, Income, and RUCA)*.

The average age of first admission was 2.4 years [standard deviation (s.d.) 3.6], and the average age at last discharge was 3.1 years (s.d. 3.8). Twenty-nine of the 91 (32%) patients received HSCT. The average age at last discharge was significantly higher for those with vs. without HSCT [mean (s.d.): 4.4 (4.4) vs. 2.5 (3.4); *p* < 0.05]. There was no significant difference in age of first admission, length of follow-up, or number of ICU days, between individuals with or without HSCT.

Thirty-two patients (35%) presented prior to 6 months of age, 10 (31%) of whom received HSCT. Thirty-five patients (39%) first presented between 6 and 36 months, 6 (17%) of whom received HSCT. Twenty-four patients (26%) presented after 36 months, 13 (54%) of whom received HSCT (Figure 1B). Individuals who presented after 36 months were more likely to receive HSCT than individuals who first presented between 6 and 36 months of life (chi-squared and ANOVA both *p* < 0.02), while all other comparisons were not significant (Tukey after ANOVA, *p* > 0.1).

To characterize survival, we evaluated for when patients were last seen, since there is not a direct measure of mortality in the database. 60 patients were not seen after 36 months of age, 14 of whom (23%) had HSCT. For the 31 patients seen after age 36 months, 15 (48%) had HSCT (Figure 1C). Of the 40 patients presumed to be alive at time of study close, 20 (50%) had HSCT (Figure 1D). Mortality and absence of HSCT were correlated (chi-squared and *t*-test *p* < 0.003). An age of last discharge greater than 36 months was shown to be correlated with the presence of HSCT (chi-squared and *t*-test *p* < 0.05). We also evaluated patient mortality by age of HSCT: less than 6 months, between 6 and 36 months and after 36 months. While older patients at the time of HSCT had higher mortality, these differences were not significant (Table 2; Figure 1E).

**Table 2:** Age of transplant (HSCT), duration of follow-up, and outcomes of ICU days and mortality.

		**Age transplant**			
**Characteristic**	**All**	** <6 mos (*N* = 8)**	**6–36 mos (*N* = 6)**	**>36 mos (*N* = 14)**	***p***
**Years followed**					>0.7
Mean (SD)	0.89 (0.80)	1.0 (0.83)	1.0 (1.1)	0.78 (0.71)	
Median [Min, Max]	0.69 [0.00, 2.9]	0.94 [0.11, 2.6]	0.51 [0.04, 2.9]	0.65 [0.0, 2.3]	
**ICU days**					>0.3
Mean (SD)	7.8 (24.8)	19.1 (44.9)	7.0 (16.2)	2.1 (4.7)	
Median [Min, Max]	0 [0, 130]	4.5 [0, 130]	0 [0, 40]	0 [0, 130]	
**Mortality**					>0.05
Alive	20 (69%)	8 (100%)	4 (67%)	7 (50%)	
Deceased	9 (31%)	0 (0%)	2 (33%)	7 (50%)	

Forty-four percent (*N* = 40) of KD patients were female and 56% (*N* = 51) were male (Table 1). 22.5% (*N* = 9) of females and 39% (*N* = 20) of males received HSCT (Figure 1F, chi-squared *p* = 0.14, *t*-test *p* = 0.08). 67.5% (*N* = 27) of females and 47% (*N* = 24) of males were determined to be deceased at the time of study close (Figure 1F, *t*-test = 0.0503, chi-squared *p* = 0.08). There were no significant differences in age of first admission [2.0 (2.9) vs. 2.7 (4.0), *p* > 0.3], age last discharge [2.8 (2.8) vs. 3.3 (4.5), *p* > 0.5], age of transplant [4.1 (4.3) vs. 3.5 (4.4), *p* > 0.7], years followed [0.8 (1.0) vs. 0.7 (0.9), *p* > 0.5], or ICU days [3.5 (8.3) vs. 5.4 (19.4), *p* > 0.5], between females and males (Table 3).

**Table 3:** KD patients evaluated by sex.

	**All (*N* = 91)**	**Female (*N* = 40)**	**Male (*N* = 51)**	***p***
**Transplant**				>0.08
No	62 (68%)	31 (77%)	31 (61%)	
Yes	29 (32%)	9 (23%)	20 (39%)	
**Age first admission**				>0.3
Mean (SD)	2.4 (3.6)	2.0 (2.9)	2.7 (4.0)	
Median [Min, Max]	0.8 [0. 17.7]	0.7 [0, 14.0]	0.9 [0.0, 17.7]	
**Age last discharge**				>0.5
Mean (SD)	3.1 (3.8)	2.8 (2.8)	3.3 (4.5)	
Median [Min, Max]	1.5 [0.1, 21.2]	1.6 [0.4, 14.2]	1.4 [0.1, 21.2]	
**Age transplant**				>0.7
Mean (SD)	3.7 (4.3)	4.1 (4.3)	3.5 (4.4)	
Median [Min, Max]	2.6 [0.0, 14.9]	3.5 [0.0, 14.0]	1.1 [0.0, 14.9]	
**Years followed**				>0.5
Mean (SD)	0.7 (0.9)	0.8 (1.0)	0.7 (0.9)	
Median [Min, Max]	0.3 [0.0, 3.9]	0.5 [0.0, 3.8]	0.3 [0.0, 3.9]	
**ICU days**				>0.5
Mean (SD)	4.6 (15.5)	3.5 (8.3)	5.4 (19.4)	
Median [Min, Max]	0 [0, 130]	0 [0, 41]	0 [0, 130	
**Mortality**				>0.05
Alive	40 (44%)	13 (33%)	27 (53%)	
Deceased	51 (56%)	27 (67%)	24 (47%)	

*Transplant (HSCT), ages, ICU days, and mortality*.

Four percent (*N* = 4) of individuals were Asian, 9% (*N* = 8) were Black, 13% (*N* = 12) were Hispanic, and 54% (*N* = 49) were White (Table 1). The remaining 20% (*N* = 18) of individuals had either multiple or unknown race or ethnicity. Fewer Black or Hispanic patients had HSCT compared to White or Asian patients, and Black and Hispanic patients had higher mortality rates, but these differences were not statistically significant (Table 4; Figures 1G,H). The age at first admission was significantly higher for Asian individuals when compared to Black, Hispanic, or White individuals [8.4 (6.3) vs. 2.1 (2.8), 1.3 (1.2) and 2.6 (3.9), ANOVA with Tukey *p* < 0.03]. The age of last discharge for Asian individuals was significantly higher when compared to Black, Hispanic, or White individuals [10.0 (7.6) vs. 2.9 (2.5), 2.2 (1.7) and 3.2 (4.0), ANOVA with Tukey *p* < 0.03]. The number of ICU days was significantly higher for Hispanic versus White individuals (ANOVA with Tukey *p* < 0.02). Seventy-five percent (*N* = 3) of Asian, 25% (*N* = 2) of Black, 17% (*N* = 2) of Hispanic, and 33% (*N* = 16) of White individuals received HSCT (ANOVA *p* > 0.3). Fifty-nine percent (*N* = 21) of White, 58% (*N* = 7) of Hispanic, 50% (*N* = 4) of Black, and 0% (*N* = 4) of Asian patients were found to be deceased (ANOVA *p* > 0.2).

**Table 4:** Race and ethnicity in KD patients.

	**Asian (*N* = 4)**	**Black (*N* = 8)**	**Hispanic (*N* = 12)**	**White (*N* = 49)**	***p***
**Age first admission**					<0.02
Mean (SD)	8.4 (6.3)	2.1 (2.8)	1.3 (1.2)	2.6 (3.9)	
Median [Min, Max]	5.9 [4.1, 17.7]	0.9 [0.0, 7.4]	0.9 [0.0, 3.2]	0.9 [0.0, 14.9]	
**Age last discharge**					<0.008
Mean (SD)	10.0 (7.6)	2.9 (2.5)	2.2 (1.7)	3.2 (4.0)	
Median [Min, Max]	6.9 [4.9, 21.2]	2.2 [0.7, 7.4]	1.6 [1.1, 3.8]	1.5 [0.1, 17.2]	
**Years followed**					>0.1
Mean (SD)	1.5 (1.3)	0.8 (0.6)	1.0 (1.3)	0.6 (0.8)	
Median [Min, Max]	1.1 [0.5, 3.5]	0.7 [0.0, 1.9]	0.5 [0.0, 3.9]	0.2 [0.0, 3.9]	
**Transplant**					>0.3
No	1 (25%)	6 (75%)	10 (83%)	33 (67%)	
Yes	3 (75%)	2 (25%)	2 (17%)	16 (33%)	
**Age transplant**					>0.3
Mean (SD)	5.4 (1.3)	0.3 (0.3)	0.1 (0.1)	4.6 (5.2)	
Median [Min, Max]	5.2 [4.1, 6.7]	0.3 [0.0, 0.5]	0.1 [0.0, 0.2]	3.2 [0.0, 14.9]	
**ICU days**					<0.05
Mean (SD)	0.25 (0.5)	3.6 (4.4)	17.6 (36.9)	2.5 (8.3)	
Median [Min, Max]	0 [0, 1]	2 [0, 10]	0.5 [0, 130]	0 [0, 41]	
**Mortality**					>0.1
Alive	4 (100%)	4 (50%)	5 (42%)	20 (41%)	
Deceased	0 (0%)	4 (50%)	7 (58%)	29 (59%)	

Seventy percent (*N* = 64) of individuals were classified as living in metropolitan areas, 7% (*N* = 6) were classified in living in micropolitan areas, 9% (*N* = 8) were classified as living in small town areas, and 4% (*N* = 4) were classified as living in rural areas based on RUCA codes (Table 1). Thirty-one percent (*N* = 20) of individuals in metropolitan areas, 33% (*N* = 2) in micropolitan areas, 37.5% (*N* = 3) in small-town, and 50% (*N* = 2) in rural areas received HSCT (Table 5; Figure 1I, ANOVA *p* > 0.8). Fifty-eight percent (*N* = 37) of individuals in metropolitan areas, 33% (*N* = 2) in micropolitan areas, 50% (*N* = 4) in small town, and 75% (*N* = 3) in rural areas were classified as deceased (Figure 1J, ANOVA *p* > 0.5). There were no significant differences in age of first admission, age last discharge, length of follow-up, transplant status, age of transplant, ICU days, or mortality based on RUCA classification (Table 5).

**Table 5:** Rural–Urban Commuting Area (RUCA) code, and median household incomes, in KD patients.

	**RUCA CODE**				
	**Urban (*N* = 64)**	**Micropolitan (*N* = 6)**	**Small Rural Town (*N* = 8)**	**Isolated Rural Town (*N* = 4)**	***p***
**Age first admission**					>0.8
Mean (SD)	2.5 (3.8)	1.9 (1.1)	3.4 (4.8)	3.3 (3.4)	
Median [Min, Max]	0.9 [0.0, 17.7]	1.9 [0.7, 3.1]	1.2 [0.1, 14.0]	2.9 [0.3, 7.2]	
**Age last discharge**					>0.5
Mean (SD)	3.1 (4.2)	2.4 (0.9)	4.1 (4.6)	4.8 (3.3)	
Median [Min, Max]	1.4 [0.0, 21.2]	2.7 [0.8, 3.3]	2.3 [0.5, 14.2]	5.4 [0.4, 14.2]	
**Years followed**					>0.3
Mean (SD)	0.7 (0.9)	0.5 (0.7)	0.7 (0.9)	1.4 (1.6)	
Median [Min, Max]	0.3 [0.0, 3.9]	0.1 [0.0, 1.7]	0.3 [0.0, 2.6]	1.0 [0.1, 3.7]	
**Transplant**					>0.2
No	44 (69%)	4 (67%)	5 (63%)	2 (50%)	
Yes	20 (31%)	2 (33%)	3 (38%)	2 (50%)	
**Age transplant**					>0.7
Mean (SD)	3.5 (4.3)	2.1 (1.5)	6.5 (7.1)	6.4 (1.3)	
Median [Min, Max]	2.3 [0.0, 14.9]	2.1 [1.0, 3.2]	5.5 [0.0, 14.0]	6.3 [5.4, 7.3]	
**ICU days**					>0.8
Mean (SD)	4.9 (17.5)	0.3 (0.8)	1.1 (3.2)	6.5 (7.2)	
Median [Min, Max]	0 [0, 130]	0 [0, 2]	0 [0, 9]	5 [0,16]	
**Mortality**					>0.5
Alive	27 (42%)	4 (67%)	4 (50%)	1 (25%)	
Deceased	37 (58%)	2 (33%)	4 (50%)	3 (75%)	
	**Median Household Incomes**				
	** <25 K** ** (*****N*** **=** **9)**	**25–50 K** ** (*****N*** **=** **48)**	**50–75 K** ** (*****N*** **=** **23)**	**>75 K** ** (*****N*** **=** **3)**	***p***
**Age first admission**					>0.1
Mean (SD)	2.8 (2.4)	2.1 (3.5)	2.8 (4.0)	6.8 (7.0)	
Median [Min, Max]	2.9 [0.1, 7.2]	0.8 [0.0, 14.9]	1.0 [0.0, 17.7]	5.6 [0.4, 14.3]	
**Age last discharge**					>0.4
Mean (SD)	3.5 (2.6)	3.0 (3.7)	3.2 (4.5)	7.0 (7.0)	
Median [Min, Max]	3.3 [0.6, 8.0]	1.8 [0.1, 17.2]	1.4 [0.4, 21.2]	6.3 [0.4, 14.3]	
**Years followed**					>0.2
Mean (SD)	0.8 (1.0)	0.9 (1.1)	0.4 (0.8)	0.2 (0.4)	
Median [Min, Max]	0.6 [0.0, 3.1]	0.5 [0.0, 3.9]	0.3 [0.0, 3.5]	0.0 [0.0, 0.7]	
**Transplant**					>0.5
No	5 (56%)	32 (67%)	16 (70%)	3 (100%)	
Yes	4 (44%)	16 (33%)	7 (30%)	0 (0%)	
**Age transplant**					>0.4
Mean (SD)	4.9 (1.9)	4.5 (5.4)	2.1 (2.3)	NA	
Median [Min, Max]	4.8 [2.9, 7.3]	2.3 [0.0, 14.9]	0.8 [0.0, 5.2]		
**ICU days**					>0.5
Mean (SD)	4.8 (8.9)	3.0 (7.2)	8.4 (27.4)	NA	
Median [Min, Max]	0 [0, 24]	0 [0, 41]	0 [0, 130]		
**Mortality**					>0.3
Alive	4 (44%)	25 (52%)	7 (30%)	1 (33%)	
Deceased	5 (56%)	23 (48%)	16 (70%)	2 (67%)	

*Median household incomes reported in $1,000 (e.g., 25 K = $25,000)*.

Ten percent (*N* = 9) of individuals had an annual median household income of < $25,000 per year, 53% (*N* = 48) had an income between $25,000 and $50,000, 25% (*N* = 23) had an income between $50,000 and $75,000, and 5% (*N* = 3) had an income >$75,000 (Table 5). Nine percent (*N* = 8) had missing median household income data. Forty-four percent (*N* = 4) of individuals with income < $25,000, 33% (*N* = 16) of individuals with income between $25,000 and $50,000, 30% (*N* = 7) with income between $50,000 and $75,000, and 0% (*N* = 0) of individuals with income >$75,000 received HSCT (Table 5; Figure 1K, ANOVA *p* > 0.5). Fifty-six percent (*N* = 5) of individuals with income < $25,000, 48% (*N* = 23) of individuals with income between $25,000 and $50,000, 70% (*N* = 16) of individuals with income between $50,000 and $75,000, and 67% (*N* = 2) of individuals with income >$75,000 were presumed to be deceased (Table 5; Figure 1L, ANOVA *p* > 0.3). There were no significant differences in age of first admission, age last discharge, length of follow-up, transplant status, age of transplant, ICU days, or mortality based on income (Table 5).

## Discussion

We report a national level, multi-center analysis of KD patients for use and outcomes of HSCT in the United States. This is the largest study of its kind for KD patients and shows disparities in the use of HSCT and in the mortality rates for patients. The small numbers of patients in subgroup analysis limited statistical significance, but showed trends, in particular of disparities for sex, race, rural/urban location, and household income.

We found that HSCT was associated with reduced mortality over the 3-year time frame of our study. However, the overall survival for KD patients who had received HSCT was below 50%. Rates of HSCT were highest in late-onset KD patients over age 36 months (54% of patients). HSCT has not been widely studied and thus is of uncertain benefit for late infantile or late-onset KD patients (9, 21), and our observations regarding older KD patients receiving HSCT does raise the concern that HSCT is being offered to patients who may have little or no benefit. Although we compared survival with or without HSCT, we were unable to determine patient quality of life, which has been reported in some studies (7), and it will be important to assess in future studies the benefit/cost ratio of HSCT for KD. A recent study suggests reclassifying KD patients with an age of symptom cutoff at 12 months into the two groups of infantile or late infantile, and that the late infantile group is more likely to benefit from HSCT, although this last point was not directly tested or reported (2). Our own data showed 100% survival after HSCT in infants <6 months of age, but survival of 67% or lower after age 6 months.

Previously, we showed that geographical location, including access to specialty leukodystrophy centers, and rural vs. urban location affect rates of diagnosis (22, 23). Here, we observed that KD patients were only reported in certain states. However, this uneven distribution could in part reflect that PHIS hospitals are not located in all 50 states; that some states have centers that specialize in HSCT for KD patients; and that some states had implemented newborn screening for KD.

An unexpected observation was that more male than female patients had HSCT. Furthermore, there was a lower mortality in male than female KD patients, suggesting that this was related to the higher proportion of males receiving HSCT. The reasons for this disparity in HSCT are not apparent for male vs. female KD patients, and could be related to age of presentation or diagnosis, speed of disease progression, or more subtle features such as perceived benefit or chance of success of HSCT by parents or caregivers.

A lower percentage of Black or Hispanic KD patients had HSCT compared to Asian or White patients. While this finding did not reach statistical significance, this could be related to the small number of patients. More Asian patients had HSCT, and they had lower mortality, compared to other racial groups. The lower mortality rates in the Asian KD patient population raises the possibility that there may be different mutation alleles or aspects of GALC enzyme activity that contribute to differences in outcomes (24). An alternative explanation to why Asian patients are coming to transplant earlier in the disease process seems unlikely since the average age of presentation of Asian patients is older than other groups. Our findings support that natural history, outcome, and treatment studies in KD should provide granular information about race and ethnicity, as well as genetic mutation type and psychosine levels and GALC activity.

We observed that patients in rural areas had the highest percentage who received HSCT (50%), but also had the highest mortality (75%) by region classification. This could indicate that patients from rural regions are inappropriately receiving HSCT, for example, even if they are already too symptomatic to receive benefit. However, we cannot exclude that patients and families from rural areas have different attitudes related to the use/non-use of HSCT. Patients from metropolitan areas, which are often underrepresented minority patients, had the lowest rate of HSCT (31%), and the second highest mortality rate (58%) by region classification. This finding is consistent with under- or late diagnosis of KD minority patients (16), but could also indicate a disparity in offering HSCT to minority patients.

It was interesting that KD patients from the lowest-income quartile (household median income < $25,000/year) had the highest HSCT percentage (44%), and that no patients from the highest quartile had HSCT. The reasons for this are uncertain, and evaluation of long-term cost burdens for families post-HSCT may be helpful to determine if middle- or higher-income families have long-term medical debts.

Limitations of this study include that it was as retrospective analysis, and only included data from PHIS hospitals. We could not account for age of first symptoms, and could only identify timing based on age at first presentation to a PHIS hospital. Our data did not track whether patients could also receive care at a non-PHIS hospital, including transplantation. Details of the *GALC* mutation, and enzyme and psychosine levels are not available in PHIS. Further, it was not possible to determine whether patients were symptomatic prior to transplantation. We could only track mortality directly if a death occurred in a PHIS hospital, which limited survival analysis. Finally, because the data was all from U.S. hospitals, aspects of our findings for KD and HSCT might be specific only to the U.S. and not generalizable world-wide.

## Conclusion

We observed disparities in the use of HSCT and in corresponding mortality rates of KD patients. The small numbers of patients in specific subgroups limited statistical significance, but had trends of lower rates of HSCT in Black and Hispanic patients. Our data also showed that there were no significant differences in ICU days or length of follow-up based on age of HSCT, suggesting that the guidelines for usage of HSCT should be evaluated. Receiving HSCT vs. not was associated with lower mortality in the time frame we analyzed, consistent with prior publications. However, the overall mortality of KD patients who received HSCT was 31%, higher than is commonly reported. This suggests that either patients who are not appropriate candidates are receiving HSCT or the efficacy of HSCT is lower than published. Our findings of disparities in treatment and outcomes emphasize the need to improve approaches to diagnosis and care for KD patients and to standardize guidelines across all centers for use of HSCT.

## Data Availability Statement

The original contributions presented in the study are included in the article/supplementary material, further inquiries can be directed to the corresponding author/s.

## Ethics Statement

The studies involving human participants were reviewed and approved by Institutional Review Boards at the University of Utah and Intermountain Healthcare. Written informed consent from the participants' legal guardian/next of kin was not required to participate in this study in accordance with the national legislation and the institutional requirements.

## Author Contributions

JB conceptualized the study. All authors designed the study, analyzed the data, drafted and edited the manuscript for intellectual content, and read and approved the final manuscript.

## Funding

GG was supported by the American Academy of Neurology Medical Student Summer Research Project. JB was supported by NIH U54NS115052.

## Conflict of Interest

JB reports serving as consultant to Bluebird Bio, Calico, Enzyvant, Denali Therapeutics, Neurogene, and Passage Bio, and on the Board of Directors of wFluidx; owning stock in Orchard Therapeutics; receiving royalties from Manson Publishing and from BioMerieux (spouse); and receiving research support from NIH and from the United Leukodystrophy Foundation. There was no role of the funders in design and conduct of the study; collection, management, analysis, and interpretation of the data; preparation, review, or approval of the manuscript; and decision to submit the manuscript for publication. The remaining authors declare that the research was conducted in the absence of any commercial or financial relationships that could be construed as a potential conflict of interest.

## Publisher's Note

All claims expressed in this article are solely those of the authors and do not necessarily represent those of their affiliated organizations, or those of the publisher, the editors and the reviewers. Any product that may be evaluated in this article, or claim that may be made by its manufacturer, is not guaranteed or endorsed by the publisher.

## Footnotes

^1^R v.4.0.2 [online]. Accessed at: www.r-project.org. Accessed April 4, 2020.

^2^Rich B. table 1 [online]. Accessed at: www.rdocumentation.org/packages/table1. Accessed April 10, 2020.

^3^Rural-Urban Commuting Area (RUCA) Codes [online]. Accessed at: www.ers.usda.gov/data-products/rural-urban-commuting-area-codes/documentation/. Accessed September 6, 2020.

^4^Larson E PD. Using RUCA Data [online]. Accessed at: https://depts.washington.edu/uwruca/ruca-uses.php. Accessed October 25, 2020.
